# Does an app designed to reduce repetitive negative thinking decrease depression and anxiety in young people? (RETHINK): a randomized controlled prevention trial

**DOI:** 10.1186/s13063-023-07295-z

**Published:** 2023-04-25

**Authors:** Julia Funk, Johannes Kopf-Beck, Edward Watkins, Thomas Ehring

**Affiliations:** 1 Department of Psychology, LMU Munich, Munich, Germany; 2 Mood Disorders Centre, School of Psychology, University of Exeter, Exeter, UK

**Keywords:** Prevention, App, Depression, Anxiety, Repetitive negative thinking, Rumination, Worry, Randomized controlled trial

## Abstract

**Background:**

The first onset of common mental health disorders, such as mood and anxiety disorders, mostly lies in adolescence or young adulthood. Hence, effective and scalable prevention programs for this age group are urgently needed. Interventions focusing on repetitive negative thinking (RNT) appear especially promising as RNT is an important transdiagnostic process involved in the development of depression and anxiety disorders. First clinical trials indeed show positive effects of preventative interventions targeting RNT on adult as well as adolescent mental health. Self-help interventions that can be delivered via a mobile phone app may have the advantage of being highly scalable, thus facilitating prevention on a large scale. This trial aims to investigate whether an app-based RNT-focused intervention can reduce depressive and anxiety symptoms in young people at risk for mental health disorders.

**Methods:**

The trial will be conducted in a sample (planned *N* = 351) of individuals aged 16–22 years with elevated levels of RNT but no current depression or anxiety disorder. In a randomized controlled between-subjects design, two versions of the app-based self-help intervention will be compared to a waiting list control condition. The full RNT-focused intervention encompasses a variety of RNT-reducing strategies, whereas the concreteness training intervention focuses on only one of these strategies, i.e., concrete thinking. The primary outcome (depressive symptoms) and secondary outcomes (anxiety symptoms and RNT) will be measured at pre-intervention, post-intervention (6 weeks after pre-intervention), and follow-up (18 weeks after pre-intervention).

**Discussion:**

This trial aims to find out whether targeting RNT via an app is an effective and feasible way of preventing depression and anxiety disorders in adolescents. Since app-based interventions are highly scalable, this trial might contribute to tackling challenges related to the increasing rates of mental health disorders among young people.

**Trial registration:**

https://www.drks.de, DRKS00027384. Registered on 21 February 2022—prospectively registered.

**Supplementary Information:**

The online version contains supplementary material available at 10.1186/s13063-023-07295-z.

## Administrative information

Note: the numbers in curly brackets in this protocol refer to SPIRIT checklist item numbers. The order of the items has been modified to group similar items (see http://www.equator-network.org/reporting-guidelines/spirit-2013-statement-defining-standard-protocol-items-for-clinical-trials/).

**Table:** 

Title {1}	Does an app designed to reduce **re**petitive negative **think**ing decrease depression and anxiety in young people? **(RETHINK):** A randomized controlled prevention trial
Trial registration {2a and 2b}.	DRKS00027384, German Clinical Trials Register https://www.drks.de. The register used for registration collects all items from the World Health Organization Trial Registration Data Set.
Protocol version {3}	15 March 2022, Original.
Funding {4}	The trial is an investigator-initiated trial, and the trial sponsor is LMU Munich, Munich Germany. Julia Funk is a fellow of the Studienstiftung des deutschen Volkes.
Author details {5a}	Julia Funk^1^, Johannes Kopf-Beck^1^, Edward Watkins^2^ & Thomas Ehring^1^ ^1^Department of Psychology, LMU Munich, Munich, Germany ^2^ Mood Disorders Centre, School of Psychology, University of Exeter, Exeter, UK
Name and contact information for the trial sponsor {5b}	Investigator-initiated trial (sponsor: LMU Munich, Munich Germany).
Role of sponsor {5c}	As this is an investigator-initiated trial, the main investigators are located at the institution serving as the trial sponsor. Only the named investigators had an influence on conduct, delivery or reporting of the research.

## Introduction

### Background and rationale {6a}

Adolescence and young adulthood bear a heightened risk for the development of mental health disorders. For example, the first onset of mood and anxiety disorders often lies in the teenage years or early twenties [1–3]. In addition to reducing the quality of life, mental health disorders can have other severe consequences, such as an increased risk for suicide attempts [4, 5] and economic costs for society [6, 7], e.g., more health care utilization, special educational needs, and a high need for family care. Thus, there is an urgent need for effective prevention and treatment of mental health problems in young people.

While traditional (face-to-face) psychological interventions have been shown to be effective in the prevention of mental health disorders, it is usually not feasible to deliver these interventions on a large scale. It has therefore been argued that Internet- and mobile-based interventions (IMIs) are a promising avenue to increase access to prevention programs [8]. In their review, Ebert et al. [8] report findings from several randomized controlled trials (RCTs) showing that IMIs decrease the risk for mental health disorders such as depression compared to waiting list control conditions; however, the current evidence is limited by a small number of existing RCTs, and studies testing IMIs in younger age groups (i.e., adolescents) are still scarce. As discussed in a recent meta-review on mobile interventions ranging from commercial mediation apps to mobile interventions based on cognitive behavioral therapy [9], the content of the interventions might crucially contribute to whether or not they have an effect on mental health outcomes. A relatively novel approach to the prevention of mental health disorders is IMIs focusing on the reduction of repetitive negative thinking (RNT, i.e., rumination and worry) [10, 11].

Prevention focusing on RNT has been highlighted as a promising strategy [12] for several reasons. First, substantial evidence suggests that RNT in the form of rumination and/or worry is an important transdiagnostic risk factor for psychopathology [13–16]. The overarching construct of RNT can be defined as repetitive thinking about one or more negative topic(s) that is experienced as difficult to control and unproductive [13, 17–20]. RNT can for example occur in the form of rumination about one’s own sad mood [21] or worry about potential future problems [22]. In longitudinal studies, RNT in the form of rumination and/or worry was found to increase the risk for a broad range of future mental health disorders including depression, anxiety disorders, and posttraumatic stress disorder [13, 15, 16, 23, 24]. Moreover, cross-sectional findings demonstrate that RNT is related to existing mental health disorders [20, 25] and indicate that RNT is involved in the maintenance of psychopathology. Importantly, experimental studies furthermore support the notion that RNT is not only an epiphenomenon or early sign of psychopathology, but causally involved in development and maintenance of mental health problems [26–28]. Of specific relevance to this trial, RNT was found to play a central role in adolescents’ mental health (for a review, see [29]). For instance, adolescent RNT was shown to prospectively predict the onset of major depression [30], explain current depressive and social anxiety symptoms [31], and mediate the relationship between infant temperament and adolescent depressive symptoms [32].

Importantly, results from a growing number of clinical trials suggest that RNT is modifiable and support the usefulness of focusing on RNT in the prevention and treatment of mental health disorders. For example, rumination-focused cognitive-behavioral therapy (RFCBT) [16, 33] is a variant of CBT that specifically targets rumination. RFCBT was originally developed as a treatment for depression but has recently been extended to treat and prevent a broader range of mental disorders by addressing transdiagnostic RNT. In RFCBT and related RNT-focused approaches, different modules are typically combined to reduce RNT as effectively as possible. These modules include identifying warning signs for RNT as well the repeated practice of different helpful habits and alternative strategies that are incompatible with RNT such as being more specific and concrete, relaxation, problem-solving, and self-compassion [33].

A series of randomized controlled trials have provided evidence for the effectiveness of RFCBT in reducing RNT and depressive symptoms as well as preventing relapse in adults and adolescents with a history of depression [34–36]. Two trials tested RFCBT as a preventive intervention for adolescents [10, 11]. In one of these trials [10], group and guided Internet RFCBT were compared to a waiting list control condition in adolescents with high levels of rumination and/or worry but no current depressive or anxiety disorder. Both RFCBT interventions significantly decreased RNT as well as sub-threshold depressive and anxiety symptoms post-intervention and over a 1-year follow-up period. Additionally, both interventions significantly reduced the 1-year incidence of major depression and generalized anxiety disorder. Cook et al. [11] replicated these findings in a sample of undergraduate university students with elevated RNT. Additionally, this trial included an arm in which participants received an unguided version of Internet RFCBT. Interestingly, unguided and guided RFCBT showed similar effects, which indicates that RFCBT has potential as a scalable online intervention.

The current trial is based on these prior studies [10, 11] and aims to extend their findings by addressing the following limitations. Firstly, prior findings suggest that RNT-focused interventions are effective in reducing sub-threshold depressive and anxiety symptoms in populations at high risk for psychopathology; however, the evidence is limited by a small number of studies. Hence, further large-scale trials are needed to test the robustness of these promising but preliminary findings. This includes testing whether the results replicate when delivering the interventions via IMIs such as scalable and easy-to-access mobile phone apps. We aim to contribute more decisive evidence based on a well-powered trial. Secondly, the efficacy of the single elements of the intervention has not yet been established. Evidence from experimental psychopathology studies [37–40] and preliminary clinical findings [41, 42] suggest that training concrete thinking could be an active ingredient of the treatment and might have potential as a stand-alone intervention. Focusing mainly on concreteness training might be particularly efficient in preventing at-risk individuals from developing more severe mental health problems; however, this has not yet been tested empirically. We aim to extend prior findings by exploring concreteness training as an alternative to a more extensive RNT-focused intervention. As both concreteness training and the full intervention have potential benefits, there is no clear rationale for the superiority of one of the two interventions. While the full intervention provides a greater variety of RNT-reducing strategies and therefore potentially more flexibility, the concreteness training intervention offers a more focused training of one strategy and hence potentially leads to better mastery of this strategy. The results from our exploratory analyses of potential differences between the two interventions could therefore yield valuable information for future studies. Finally, prior trials mostly tested the effects of RFCBT and related interventions on depressive symptoms and generalized anxiety symptoms. However, social anxiety symptoms were also found to be related to RNT [43–45] and thus might decrease after RNT-focused interventions. This is especially relevant for trials in younger age groups as social anxiety is prevalent in adolescents [46]. Thus, we will include social anxiety as an additional outcome measure.

## Objectives {7}

The primary aim of this trial is to compare an app-based self-help RNT-focused preventive intervention to a waiting list control condition in a sample of adolescents and young adults who are at risk for developing a depressive or anxiety disorder due to high levels of RNT. To explore active ingredients of the treatment, two versions of the app-based intervention will be tested against the control condition, namely the more extensive full RNT-focused intervention and concreteness training as a stand-alone intervention. The secondary goal of the trial is to compare the effects of the two interventions and thereby inform future studies seeking to dismantle effective components of RNT-focused interventions.

As RNT is a risk factor predicting future psychopathology (e.g., depression) and we will test individuals with high levels of RNT at the beginning of the trial, we expect psychopathological symptoms to increase or remain constant in the waiting list control group. In contrast, we assume that both interventions will have beneficial effects in that they decrease sub-threshold psychopathological symptoms. Specifically, we will test the following hypotheses. First, we predict that both app-based interventions will reduce depressive symptoms (primary outcome) relative to the waiting list control condition. Second, we hypothesize that both preventive interventions will reduce scores on our secondary outcome measures for the risk factor RNT as well as generalized anxiety symptoms and social anxiety symptoms. Since decreases in sub-threshold symptoms and RNT are precursors to prevention but no direct test of preventive effects, we will explore whether the interventions decrease the probability of meeting criteria for depression and anxiety disorders as indexed by cut-offs on self-report measures. Finally, the two interventions will be compared regarding their effect on the primary and secondary outcomes. As concreteness training and the full RNT-focused intervention have not been tested in the same trial prior to this study, we do not have any predictions regarding whether one of the apps will be more efficacious.

## Trial design {8}

This trial employs a superiority, three-arm parallel-group randomized controlled design, comparing two app-based interventions to a waiting list control condition. Participants will be allocated randomly (in a 1:1:1 ratio) to receive the full-RNT focused intervention via mobile phone app or the concreteness training intervention via mobile phone app or to wait for 18 weeks before being offered one of the two app versions. Thus, the three trial arms are as follows: (1) the full RNT-focused intervention, (2) the concreteness training intervention, and (3) the waiting list control condition.

## Methods: participants, interventions, and outcomes

### Study setting {9}

We plan to recruit 351 adolescent and young adult participants within Germany. Participants will be recruited via advertisements on social media (e.g., on Facebook, Instagram, Twitter), mailing lists, and newsletters, as well as other circulars and noticeboards within willing schools and universities. The study will be conducted online using the survey platform *Research Electronic Data Capture* (*REDCap*) [47] for assessments and the services of the software developer *m-Path* [48] for the app-based interventions.

### Eligibility criteria {10}

Eligible participants will be (1) aged 16 to 22 years, (2) living in Germany, (3) having regular access to an iOS or android smartphone, and (4) showing sum scores at or above the 75th percentile on a measure of RNT, either ≥ 40 on the *Ruminative Response Scale* (*RRS*) [49] or ≥ 50 on the *Penn State Worry Questionnaire* (*PSWQ*) [50]. As the current trial is designed to be a prevention trial (and not a treatment trial), participants meeting the criteria for major depression, generalized anxiety disorder, and social anxiety disorder at the beginning of the trial are not eligible for participation. Probable diagnoses will be determined by standard cut-offs on self-report measures, i.e., sum scores > 9 on the *Patient Health Questionnaire-9* (*PHQ-9*) [51], sum scores > 9 on the *Generalized Anxiety Disorder-7 Questionnaire* (*GAD-7*) [52], and sum scores > 35 on the *Social Interaction Anxiety Scale* (*SIAS*) [53]. In addition, participants currently receiving psychotherapy will be excluded from participation.

### Who will take informed consent? {26a}

Basic study information including the eligibility criteria can be found on all materials used for recruitment (flyers, social media posts, etc.). Potential participants who are interested in the study will be directed to the digital survey platform REDCap. They can either access the link to the study via the recruitment material or write an e-mail to the study team, who will then send them the link. In REDCap, participants will be provided with the study information sheet, which contains more detailed information about the study. They will be asked to provide informed consent by electronically signing the online informed consent form and entering their name, birth date, and contact information (e-mail and phone number). Underaged participants will be asked for additional parental informed consent. Only participants who provided informed consent and underaged participants whose parents (legal guardians) additionally provided informed consent will be forwarded to the eligibility screening for the study.

### Additional consent provisions for collection and use of participant data and biological specimens {26b}

n/a, explanation: The data collected within this trial will not be used for purposes that are separate from the trial. Biological samples will not be collected.

### Interventions

#### Explanation for the choice of comparators {6b}

We chose to have a waiting list control condition as the comparator because there are no standard preventive interventions for the specific target group of this study to which the full RNT-focused intervention and the concreteness training intervention could be compared to. In addition, as the study investigates an intervention aimed at prevention, it is crucial to have a control condition representing the symptom development without any intervention provided. Participants in the waiting list control condition will be offered the option to download one of the two app-based interventions and use them for 6 weeks after they completed the study.

#### Intervention description {11a}

See Table 1 for an overview of the interventions and Supplementary Material for a detailed description of different modules and key elements of the interventions.

**Table 1:** Modules and key elements of the app-based interventions

Full RNT-focused intervention		Concreteness training intervention	
Module	Key elements	Module	Key elements
Identifying triggers of RNT and stress	*- Challenge:* Personal warning signs	Identifying triggers of RNT and stress	*- Challenge:* Personal warning signs
	*- Mood tracker*		
Concreteness Training	*- Challenge*: Abstract versus concrete thinking	Concreteness Training	*- Challenge*: Abstract versus concrete thinking
	*- Tool:* Concrete thinking		*- Tool*: Concrete thinking
Engaging in Opposite Action	*- Tool:* Opposite Action		
Self-Compassion	*- Challenge:* Kind versus unkind self-talk		
	*Tool: Kind self-talk*		
Mindfulness	*- Tool: Mindfulness*		
Setting Priories	*- Tool:* Setting priorities		
Transfer to Everyday Life	*- If–then-plans*	Transfer to Everyday Life	*- If–then-plans*

Both interventions will be delivered via a self-help app

*RNT* Repetitive negative thinking

##### Full RNT-focused intervention

The full RNT-focused intervention was developed based on principles of RFCBT [16, 33]. Similar app-based interventions grounded in RFCBT are currently also being evaluated as part of two prevention trials related to the current trial [54, 55].

The app consists of the following modules: psychoeducation on RNT and different strategies to reduce RNT, identifying personal triggers of RNT and stress, concreteness training, engaging in opposite actions, relaxation/mindfulness-based exercises, self-compassion, setting priorities to cope with stress-related worries, tracking current emotions and repetitive thoughts, and making specific if–then-plans to apply the acquired strategies in every-day life.

The app is organized into different sections. The section *knowledge* contains psychoeducation on RNT and different strategies to reduce RNT. The section *challenges* contains exercises to compare different less helpful (RNT-related) and more helpful (RNT-reducing) styles of reacting to difficult situations, for example abstract vs. concrete thinking or kind vs. unkind self-talk. The section *tools* consists of exercises that facilitate transfer of the different helpful, RNT-reducing strategies to everyday life. The section *mood tracker* offers the possibility to track current emotions and repetitive thoughts in day-to-day life. In the section *if–then-plans*, participants can make specific plan to use the acquired strategies in their daily lives.

Participants will be able to choose activities from these different sections and adjust the intervention to their current needs. They will be instructed to use the app as consistently as possible over a period of 6 weeks. Additionally, the app will automatically display pop-up reminders (three to four per week) to complete certain exercises (i.e., particular challenges and tools) in the app. The number of pop-up reminders (three per each tool and three per each challenge) is balanced across the different exercises so that participants will receive the same number of reminders for each of the tools and challenges.

To make the app as user-friendly and helpful as possible, it combines animations, videos, audio exercises, explanatory texts, and multiple choice and open-question formats. Additionally, the app asks participants to indicate how helpful the exercise was after completion of each challenge or tool and gives conditional feedback and tips based on participants’ answers.

The app was designed in cooperation with the software developer m-Path and is compatible with iOS and Android.

##### Concreteness training intervention

This intervention app focuses on one module of the full RNT-focused intervention—training concrete thinking. Basic design, layout, and organization of this app are the same as in the full RNT-focused intervention app. However, the content of concreteness training app is leaner and more focused. The section *knowledge* contains psychoeducation on RNT and concrete thinking as a strategy to reduce RNT. The section *challenges* provides exercises to compare unhelpful, abstract RNT-related thinking and helpful, concrete thinking. The section *tools* consists of exercises that facilitate the transfer of helpful, concrete thinking to everyday life. In the section *if–then-plans*, participants can make specific plans to use the acquired strategies in their everyday lives. The elements acknowledging triggers for RNT in *challenges* and the section *if–then-plans* are included in the concreteness training intervention to facilitate the transfer of helpful concrete thinking to participants everyday life—particularly to situations that typically elicit RNT. As there are less different challenge and tools in the concreteness training app than in the full-RNT-focused app, participants in the concreteness training condition will receive a smaller number of pop-up reminders in total, which is in line with the goal to test concreteness training as a more time-efficient alternative to the full intervention.

#### Criteria for discontinuing or modifying allocated interventions {11b}

n/a, explanation: The use of the app for participants of the current trial is clearly labeled as self-help and non-equivalent to psychotherapy for mental health disorders. Potential participants fulfilling the criteria for major depression, generalized anxiety disorder, or social anxiety disorder will be excluded from participation and provided with information, guidance including to consult with their general practitioner (or equivalent), weblinks, and telephone numbers for help and support. There is no indication that the interventions cause harm or any unwanted side effects; similar interventions have rather been shown to have positive effects on mental health among participants with depressive and/or anxiety symptoms below the clinical threshold but a tendency towards RNT [10, 11]. Therefore, there are no criteria for discontinuing or modifying the intervention.

#### Strategies to improve adherence to interventions {11c}

Participants will be told that the apps are most effective when consistently used several times during the study. Additionally, both apps send push-up notifications reminding participants to complete certain exercises (i.e., challenges and tools) in the app. The use of the apps will be monitored including the number of times the app is used, and the number of times single challenges and tools have been completed/used in the app. A minimum intervention dose for the intervention conditions will be defined a priori, based on the rationale that active ingredients of the treatments are learning new concepts (completing *challenges*) and practicing new skills (completing *tools*).

#### Relevant concomitant care permitted or prohibited during the trial {11d}

Individuals who are currently receiving psychotherapy cannot participate in the trial.

#### Provisions for post-trial care {30}

n/a, explanation: There is no anticipated harm and compensation for trial participation. Since there are no established interventions recommended for the target population of this trial, there will be no ancillary and post-trial care.

### Outcomes {12}

Outcomes will be assessed at three measurement timepoints: pre-intervention (i.e., pre-randomization), post-intervention (6 weeks after pre-intervention), and follow-up (18 weeks after pre-intervention).

#### Primary outcome

The primary outcome for this trial will be the sum score on the 30-item *Inventory of Depressive Symptomatology* (*IDS*) [56] at post-intervention. The IDS is a well-established measure of depressive symptoms [57].

#### Secondary outcomes

Secondary outcomes include post-intervention sum scores on the following measures: the *Generalized Anxiety Disorder Questionnaire-IV* (*GADQ-IV*) [58], a 10-item questionnaire assessing generalized anxiety symptoms; the 17-item *Social Phobia Inventory* (*SPIN*) [59] measuring social anxiety symptoms; the 22-item *Ruminative Response Scale* (*RRS*) [49], a well-established measure of depressive rumination; the *Penn State Worry Questionnaire* (*PSWQ*) [50], a 16-item scale assessing pathological worry; and the *Perseverative Thinking Questionnaire* (*PTQ*) [17], a validated 15-item questionnaire measuring process features of RNT such as repetitiveness and intrusiveness of thoughts regardless of typical thought content.

Further measures that will be administered at all three measurement timepoints are as follows: the 9-item *Patient Health Questionnaire-9* (*PHQ-9*) [51], a self-report measure for diagnosing major depression; the 7-item *Generalized Anxiety Disorder-7 Questionnaire* (*GAD-7*) [52], a self-report measure for generalized anxiety disorder; and the 20-item *Social Interaction Anxiety Scale* (*SIAS*) [53], a self-report questionnaire for diagnosing social anxiety disorder.

Demographic data and additional health-related data such as current psychotherapy and current medication will only be assessed at baseline (pre-intervention and randomization).

### Participant timeline {13}

**Fig. 1:**
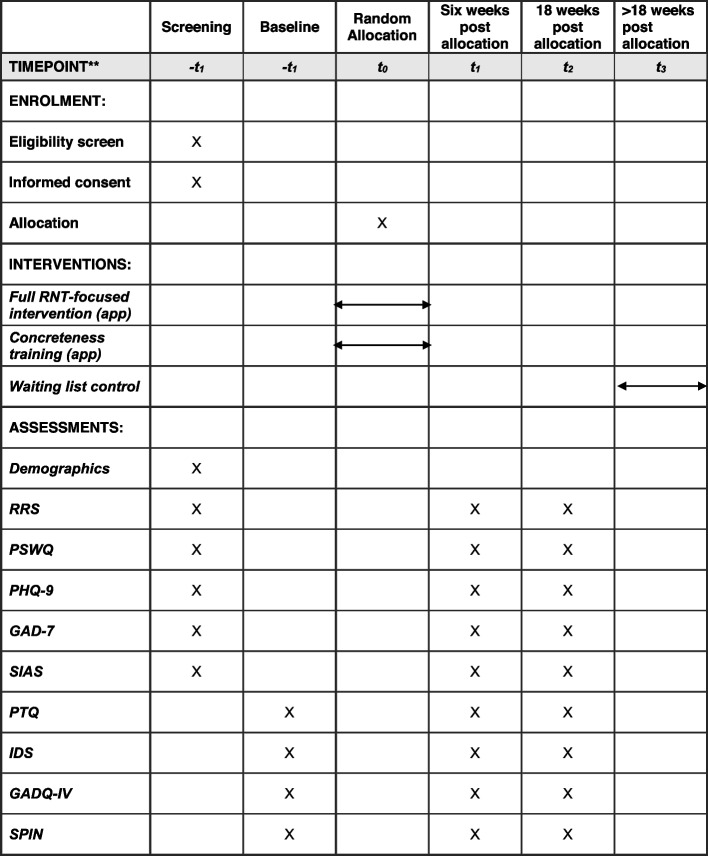
Schedule of enrolment, interventions, and assessment

**Fig. 2:**
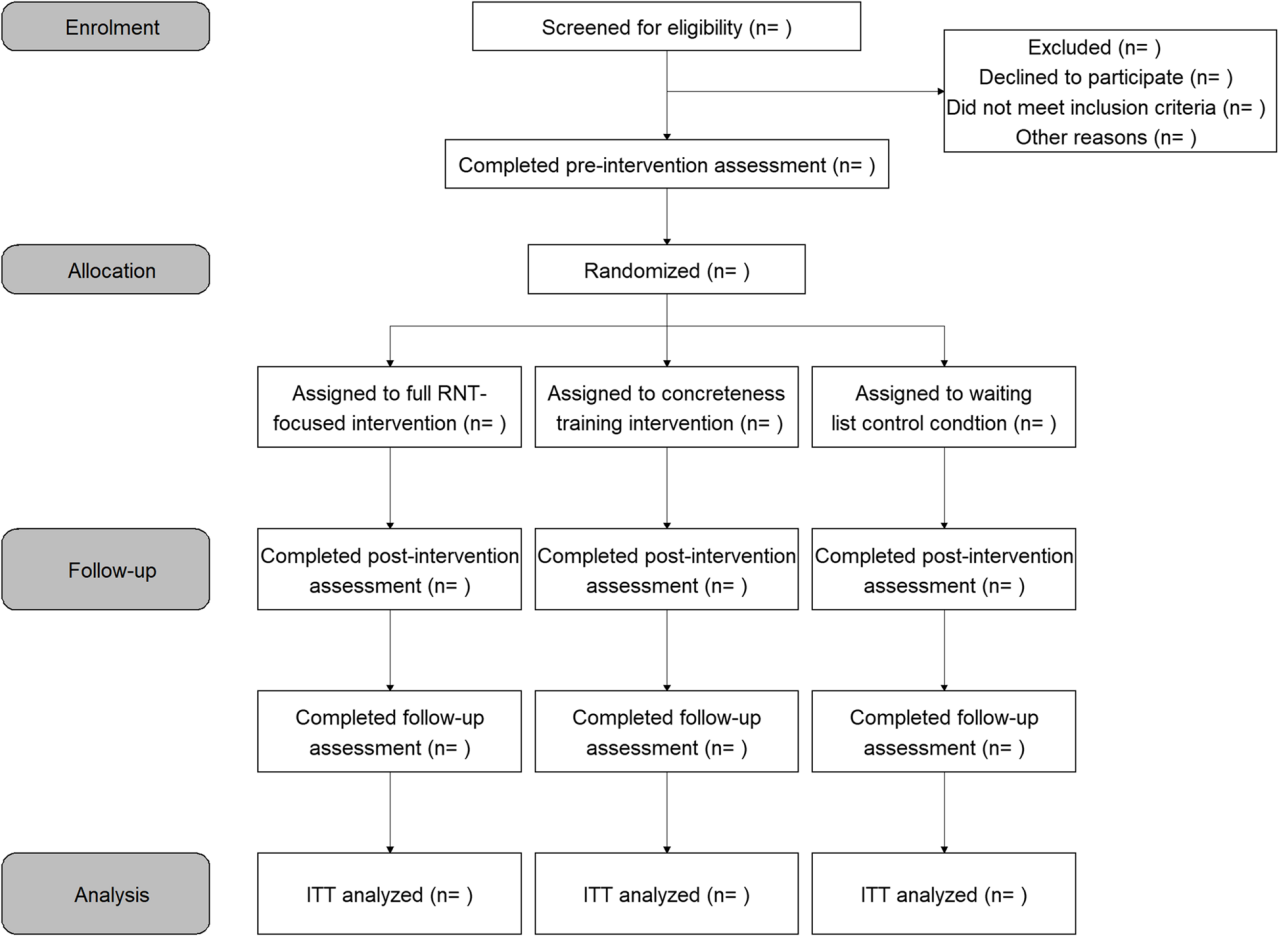
CONSORT flow diagram

Eligible participants will complete the pre-intervention assessment including measures for the primary outcome (depressive symptoms) and secondary outcomes (generalized anxiety symptoms, social anxiety symptoms, RNT) of the study. Consequently, participants will be randomly allocated to one of the three trial conditions. Participants in both active conditions (conditions (1) and (2)) will use the respective app for 6 weeks. Primary and secondary outcomes will be measured again at post-intervention (6 weeks after pre-intervention) and follow-up (18 weeks after pre-intervention). All participants will provide informed consent prior to data acquisition, randomization, and use of intervention apps. For an overview of the study procedure, see Figs. 1 and 2.

### Sample size {14}

We conducted a power analysis in G*Power [60] based on the minimal clinically important difference (MICD) in depressive symptoms (primary outcome). Measures of depressive symptoms have an established MCID of *d* = 0.48 [61], which also provided the basis for the power analysis in other recent trials testing app-based prevention [54]. Using this effect size, with 90% power and an alpha level of 0.05, the sample size required for a 2-arm two-sided comparison at post-intervention would be 93 participants per arm. To account for 20% expected dropout at post-intervention, a total sample size of 351 (117 per trial arm) will be needed to test whether the two app-based interventions (the full RNT-focused intervention and the concreteness training intervention) significantly reduce depressive symptoms relative to the waiting list control condition. The required sample size was calculated for a two-arm comparison (even though the study has three arms) as we do not have any a priori hypotheses regarding whether one of the apps will be more efficacious.

### Recruitment {15}

Participants will be recruited via advertisements on social media (e.g., on Facebook, Instagram, Twitter), mailing lists, and newsletters, as well as other circulars and noticeboards within willing schools and universities. School psychologists of willing schools will be contacted and provided with detailed information about the trial to support recruitment in single schools. As an incentive to participate, participants will have the opportunity to take part in a lottery after they completed the study. Alternative to this, participants studying psychology or a related subject at LMU Munich can receive partial course credit for completing the study.

## Assignment of interventions: allocation

### Sequence generation {16a}

Participants will be randomized (in a 1:1:1 ratio) to the three trial arms. Randomization will be conducted independently using pre-generated computerized allocations based on blocking with variable block sizes. As many of the study outcomes such as depressive symptoms and RNT are known to be distributed unequally across genders [62, 63], randomization will be stratified according to gender (male, female, non-binary).

### Concealment mechanism {16b}

Randomization will be conducted in REDCap based on a pre-generated randomization table applying block randomization and stratification by gender. Allocation concealment will be ensured, as the allocation code will not be visible for the study team before a participant has been assigned to one of the treatment conditions.

### Implementation {16c}

The randomization table providing the basis for the computerized allocation in REDCap will be created by an independent statistician outside the study team. Enrolment and the generation of the allocation code will be automatized via REDCap and hence cannot be influenced by the study team monitoring data collection.

## Assignment of interventions: blinding

### Who will be blinded {17a}

The analysis team will be blind to the trial condition. The variable condition will be masked by someone outside the analysis team before the analysis.

### Procedure for unblinding if needed {17b}

n/a, explanation: There is no blinding during data collection and thus no procedure for unblinding.

#### Data collection and management

### Plans for assessment and collection of outcomes {18a}

All assessments (pre-intervention, post-intervention, follow-up) will take place online. Data will be collected automatically via the survey platform REDCap. Only the following validated questionnaires will be used to measure the outcomes of this trial.

#### Questionnaires measuring RNT

Ruminative Response Scale (RRS): The RSS [49] (German version [64]) is a 22-item scale measuring the frequency with which respondents think about their depressive symptoms. Items such as “When I feel sad or down, I think about a past situation and wish it had gone better” are rated on a 5-point Likert scale ranging from “almost never” to “almost always”. The RRS has been shown to have good internal consistency, test–retest reliability, and high construct validity [65].

Penn State Worry Questionnaire (PSWQ): The PSWQ [50] (German Version [66]) is a 16-item questionnaire assessing the frequency, intensity, and uncontrollability of worry. Respondents are asked to rate the items such as “Many situations make me worry” on a 5-point Likert scale ranging from “not typical at all of me” to “very typical of me”. The PSWQ demonstrated high internal consistency as well as good convergent and discriminative validity [50].

Perseverative Thinking Questionnaire (PTQ): The PTQ [17] (which will be used in a German translation by the authors of the questionnaire) is a 15-item questionnaire assessing an individual’s general tendency towards repetitive negative thinking. Unlike the PSWQ and the RRS, the PTQ measures process features of RNT rather than specific thought contents of RNT. Items such as “Thoughts intrude into my mind” are rated on a 5-point Likert scale ranging from “never” to “almost always”. The PTQ demonstrated excellent internal consistency as well as high construct validity [17].

#### Questionnaires measuring depressive and anxiety symptoms

Inventory of Depressive Symptomatology (IDS): The IDS [56] is a 30-item questionnaire measuring depressive symptoms. For the current trial, a German translation of the IDS [67] will be used. Participants are asked to read statements about different depressive symptoms (e.g., “feeling sad”) and chose one of four statements (e.g., “I do not feel sad”, “I feel sad less than half the time”, “I feel sad more than half the time”, “I feel sad nearly all of the time”), which describe their state during the past 7 days most accurately. The IDS demonstrated excellent internal consistency in sub-clinical sample with Cronbach’s *α* ranging between 0.92 and 0.94 and good internal consistency in samples with current depression with Cronbach’s α ranging between 0.76 and 0.82 [56]. High correlations between the IDS and other measures of depressive symptoms indicate good construct validity [56].

Social Phobia Inventory (SPIN): The SPIN [59] (which will be used in a German translation [68]) is a 17-item measure of social anxiety symptoms. Participants are asked to rate items like “I am bothered by blushing in front of people” on a 5-point Likert scale ranging from “not at all” to “extremely”. The SPIN was shown to have good internal consistency, test–retest reliability, and high construct validity [59].

Generalized Anxiety Disorder Questionnaire-IV (GADQ-IV): The GADQ-IV [58] (which will be administered in a German translation [69]) is a 10-item scale assessing the presence and intensity of generalized anxiety disorder symptoms, such as the excessiveness and uncontrollability of worry and related physical symptoms. The answer formats are dichotomous yes–no questions for detecting the presence/absence of different symptoms and 9-point Likert scales ranging from “none” to “very severe” to assess functional impairment and subjective distress through symptoms. The GADQ-IV demonstrated high test–retest reliability as well as good convergent and discriminant validity [58].

#### Self-report questionnaire measures of probable psychiatric diagnoses

Patient Health Questionnaire-9 (PHQ-9): The PHQ-9 [51] (German version [70]) is a 9-item questionnaire commonly used for screening for depression. Respondents are asked to rate how much they have been bothered by problems such as “little interest or pleasure in doing things” on a 4-point Likert scale ranging from “not at all” to “nearly every day”. The PHQ-9 was shown to have good psychometric properties and there is high agreement between PHQ diagnoses of depression and those of independent mental health professionals [51].

##### Generalized Anxiety Disorder-7 Questionnaire (GAD-7)

The GAD-7 [52] (German version [71]) is a 7-item questionnaire commonly used for screening for generalized anxiety disorder. Participants are asked to rate how much they have been bothered by problems such as “Feeling afraid as if something awful might happen” on a 4-point Likert scale ranging from “not at all” to “nearly every day”. The GAD-7 was found to have good psychometric properties and validity as a screener for generalized anxiety disorder [52].

Social Interaction Anxiety Scale (SIAS): The SIAS [53] (German version [72]) is a 20-item scale assessing anxiety in social interaction and being observed by others. Respondents are asked to rate items such as “I get nervous if I have to speak with someone in authority (teacher, boss, etc.)” on a 5-point Likert scale ranging from “not at all” to “extremely”. The SIAS was shown to have good psychometric properties [53] and to be a valid screener for social anxiety disorder [73].

### Plans to promote participant retention and complete follow-up {18b}

To promote participant retention, participants can only receive reimbursement for their participation (either partial course credit or taking part in the lottery and having the opportunity to win an Amazon voucher) when they completed all three assessments of the study (pre-intervention, post-intervention, follow-up). To promote retention in the control group, the trial has a waiting list control condition, in which participants can download one of the two intervention apps after they completed the study.

### Data management {19}

Primary data will be collected via the software REDCap, which is running on secure, encrypted faculty servers of the LMU Munich. Metadata about the app use (frequency, which different parts of the app have been used) will be collected via the software m-Path. Pseudonymized data will be extracted to a secure, encrypted internal study drive. As data will be collected automatically via online platforms specifically designed for research purposes, high data quality will generally be guaranteed. Data will be collected and stored in a pseudonymized manner whenever possible (for the management of personal data see item 27—confidentiality).

### Confidentiality {27}

Personal data of potential and enrolled participants (name, e-mail address, and phone number) will only be collected so that participants can be contacted throughout the trial (e.g., receive the links to the different assessments). Personal data of parents (name and e-mail address of both parents/other legal guardians) will only be collected for underaged participants in order to obtain parental informed consent in accordance with German law. Personal data will be collected via the software REDCap, which is running on encrypted, secure faculty servers of the LMU Munich and will—at no point—be saved outside of the REDCap system. Only the study team (the first author of this paper and master students involved in data collection and in case of adverse events licensed clinical psychologists within team) will have access to the personal data as they need it to manage and monitor data collection or to react to adverse events. Everyone with access to the personal data has to sign an agreement stating that personal data must not be used for any other purpose than managing and monitoring data collection. For all further purposes (including logging into the m-Path app), participants will receive a pseudonym. After data collection for this study is finished, all personal data will be deleted so that the final dataset is completely anonymized. Potential participants will be informed in detail about data management and data protection in this trial before they take informed consent. The data protection and management procedures have been approved by the data protection officer of LMU Munich.

### Plans for collection, laboratory evaluation, and storage of biological specimens for genetic or molecular analysis in this trial/future use {33}

n/a, explanation: No biological specimens will be collected as part of this study.

## Statistical methods

### Statistical methods for primary and secondary outcomes {20a}

#### Primary analyses

A linear mixed-effects model will be computed to analyze whether the two versions of the app reduced depressive symptoms (sum score on the IDS, primary outcome variable) relative to the control condition. In the model, the effect of condition (full RNT-focused intervention, concreteness training intervention, waiting list control condition), time (pre-intervention, post-intervention), and condition × time interaction will be tested. Simple slope tests will be conducted to determine whether (a) the full RNT-focused intervention significantly reduced depressive symptoms relative two the waiting list control condition and (b) the concreteness training intervention significantly reduced depressive symptoms compared to the waiting list control condition.

#### Secondary analyses

The primary analyses will be repeated for the secondary outcomes depressive rumination (RRS sum score), worry (PSWQ sum score) content-independent RNT (PTQ sum score), generalized anxiety symptoms (GADQ-IV sum score), and social anxiety symptoms (SPIN sum score), respectively. *P-*values will be adjusted using Holm’s procedure [74] to reduce the chance of finding false positives due to multiple outcome variables.

#### Exploratory analyses

Comparison of the two active conditions: As described in the section objectives {7}, we do not have any a priori predictions about the superiority of one of the two app-based interventions over the other intervention. To explore potential differences in the outcome variables at post-intervention between the two active conditions, we plan to conduct a Bayesian analysis. In contrast to frequentist analyses, in which the absence of a significant effect cannot be considered as proof for the null hypothesis, in Bayesian analyses, the strength of evidence for two competing hypotheses can be assessed [75, 76]. Hence, this approach is suitable to objectively explore potential differences between experimental conditions when there are no a priori hypotheses as it also allows to express with how much certainty there are no differences between the conditions.

Effects at follow-up: All analyses will be adapted to test whether the predicted effects and potential differences between the two active conditions extend to the follow-up timepoint.

Effects on diagnoses: Logistic regression analyses will be conducted to explore whether the app-based interventions decrease the probability of fulfilling the criteria (as indexed by standard cut-offs on self-report questionnaires) for a depressive episode, generalized anxiety disorder, and social anxiety disorder at post-intervention and/or follow-up.

### Interim analyses {21b}

A full, detailed statistical analysis plan (SAP), including plans for any interim analysis, subgroup analysis, and sensitivity analysis of the primary outcomes, will be prepared and finalized before the analysis. The SAP will be made available over the platform where the study was registered (German Clinical Trials Register https://www.drks.de, study ID: DRKS00027384) before the end of recruitment.

### Methods for additional analyses (e.g., subgroup analyses) {20b}

Methods for additional analyses will also be included in the detailed analysis plan that will be finalized before the analysis.

### Methods in analysis to handle protocol non-adherence and any statistical methods to handle missing data {20c}

Missing data will be inspected and handled via full information maximum likelihood (FIML). The analyses will be intention-to-treat (ITT) [77] analyses.

### Plans to give access to the full protocol, participant-level data, and statistical code {31c}

Anonymized datasets arising from this trial as well as statistical code will be made available after the primary outcomes of this trial are published to researchers and other groups on request and via the Open Science Framework repository (https://osf.io/).

## Oversight and monitoring

### Composition of the coordinating centre and trial steering committee {5d}

As this is an online trial, in which the digital assessments and interventions will be conducted in an automatized manner, the responsibilities of the study team will be restricted to monitoring the automatic data collection and directing participants to further support/treatment options if needed. Participants will be informed that they can contact the study team anytime via the study e-mail if they have questions about the study or feel seriously distressed and need further information about support/treatment options. The study team consists of Julia Funk as lead investigator and Johannes Kopf-Beck as well as Thomas Ehring in supervisory roles. Several master-level students of psychology will assist in the running of the project.

### Composition of the data monitoring committee, its role, and reporting structure {21a}

n/a, explanation: Since this is a trial with a waiting list control condition (and not a placebo intervention control condition), blinding of participants and personnel is not possible. Therefore, the establishment of a data monitoring committee, which is normally installed in masked trials to supervise adverse events and potential relations to the experimental treatment conditions, is not necessary.

### Adverse event reporting and harms {22}

Adverse events such as suicidality, self-harm, or extreme increases in depressive or anxiety symptoms and other unintended effects of trial interventions or trial conduct will be assessed through standardized questions about suicidality and symptoms at the pre-intervention, post-intervention, and follow-up assessment, plus spontaneously reported feedback from participants (e.g., via the study e-mail). If there are indications for adverse events, the study team will—according to a risk management protocol—contact licensed clinical psychologists associated with the study, who will then call the respective participants to confirm the occurrence of an adverse event and act accordingly, i.e., direct the participant to psychiatric care. Adverse events will be reported on a regular base to the ethics committee of the faculty of psychology and learning sciences of LMU Munich.

### Frequency and plans for auditing trial conduct {23}

n/a, explanation: As the interventions in the current trial will be administered via app in an automatized manner and not by study personnel, such plans are not necessary.

### Plans for communicating important protocol amendments to relevant parties (e.g., trial participants, ethical committees) {25}

For all modifications, we will seek approval of the ethics committee of the faculty of psychology and learning sciences of LMU Munich. Additionally, in case of modifications, the registration on the trial registry DRKS will be updated. Furthermore, the recruitment material and study information sheet will be updated if the modifications include changes to the eligibility criteria or the study procedure.

#### Dissemination plans {31a}

Results of this trial will be made available to the scientific community by publishing the findings in scientific journals as well as to the broader public by communicating the results on websites and social media or in the press.

## Discussion

Adolescence (and young adulthood) is a particularly vulnerable phase, in which many mental health disorders such as mood and anxiety disorders have their first onset [1, 2]. Considering recent increases in adolescent mental disorders related to the coronavirus pandemic [78], there is an urgent need for effective and scalable prevention programs for this age group. The current trial aims to contribute to a better understanding of how effective prevention of mental health disorders in young people can be achieved on a large scale by testing a preventive approach that combines two promising strategies.

Firstly, the intervention(s) tested in this trial will be delivered via a mobile phone app. In contrast to in-person prevention programs guided by a therapist or trainer, app-based interventions can be made widely accessible especially in younger age groups since the majority of young people uses mobile devices [79]. In addition to high scalability and convenience, app-based approaches appear ideal for establishing helpful and functional habits in daily life. An app is always at hand via smartphone and thus ideal for integrating behavioral changes in daily life and providing support in coping with problems whenever needed. Despite these potential benefits and the growing number of mental health apps [80], evidence for their effectiveness is still limited due to a small number of rigorous RCTs investigating the effects of apps on mental health outcomes. The current trial, in line with other currently running online RCTs [54, 81], aims to fill this research gap by comparing the effects of potentially highly scalable app-based interventions to a waiting list control group.

Secondly, instead of narrowly focusing on a risk factor for a single mental health disorder, the preventive intervention(s) tested in the current trial target(s) a *transdiagnostic* risk factor, which plays a role in various types of psychopathology. Transdiagnostic risk factors have been highlighted as useful targets for treatment and especially prevention of mental health disorders [82, 83]. Unlike intervention protocols for specific mental health disorders, transdiagnostic approaches take issues such as the frequent comorbid presentation of different disorders into account and help to tackle underlying causal processes instead of merely focusing on symptom reduction [84]. This broader and more causally oriented focus is particularly purposeful for the prevention of mental health disorders as the target population for prevention by definition does not meet the full criteria for a specific mental health disorder. Moreover, longitudinal research and theoretical models suggest that certain psychological variables do not only increase the risk for developing one specific but several different mental health disorders (i.e., multifinality) [85]. Transdiagnostic preventive approaches aim to reduce these dysfunctional psychological processes before they manifest into more severe mental health problems and might therefore be especially effective as they decrease the risk of different mental health disorders.

The preventive intervention(s) tested in the current study focus(es) on RNT, which is an important transdiagnostic process. Next to the general benefits of transdiagnostic approaches mentioned above, focusing on RNT might especially effectively increase psychological resilience among adolescents as there is growing evidence that RNT contributes to the development of mental health disorders particularly in this age group [29]. Additionally, RNT is easily delectable through self-report questionnaires [17, 86], and importantly, a number of studies found that RNT is modifiable by psychological interventions—specifically in adolescents [10, 11, 36].

Finally, the current trial aims to contribute to a better understanding of the active ingredients in RNT-focused interventions. There are several possible strategies, which can help to reduce RNT. In existing RNT-focused approaches, such as RFCBT [33] and metacognitive therapy [87], usually several of these strategies are combined to reduce RNT as effectively as possible. However, a recent review highlighted the importance of investigating active ingredients and mechanisms of change in psychological interventions in order to better understand for which individuals the interventions might work best and how interventions can be made more effective and cost-efficient [88]. The current study aims to contribute to tackling this challenge by employing a component design and testing an especially promising element of the full treatment (concreteness training) in a separate trial arm as a stand-alone intervention.

## Trial status

Protocol version number and date: 15 March 2022, original.

Date when recruitment began: 01 March 2022.

Approximate date when recruitment will be completed: 31 July 2023.

## Supplementary Information

**Additional file 1.** 

## Data Availability

All authors of this paper and master students writing their thesis within the project will have access to the final dataset set. Anonymized datasets arising from this trial as well as statistical code will be made available after the primary outcomes of this trial are published to researchers and other groups on request and via an open data repository.

## Abbreviations

GAD-7: Generalized Anxiety Disorder-7 Questionnaire
GADQ-IV: Generalized Anxiety Disorder Questionnaire-IV
FIML: Full information maximum likelihood
IDS: Inventory of Depressive Symptomatology
IMI: Internet- and mobile-based intervention
ITT: Intention-to-treat
MCID: Minimal clinically important difference
PHQ-9: Patient Health Questionnaire-9
RCT: Randomized controlled trial
REDCap: Research Electronic Data Capture
RETHINK: App-based prevention designed to reduce repetitive negative thinking
RFCBT: Rumination-focused cognitive-behavioral therapy
RNT: Repetitive negative thinking
RRS: Ruminative Response Scale
PSWQ: Penn State Worry Questionnaire
PTQ: Perseverative Thinking Questionnaire
SIAS: Social Interaction Anxiety Scale
SPIN: Social Phobia Inventory
SAP: Statistical analysis plan
