# Supplementary material for: Does an app designed to reduce repetitive negative thinking decrease depression and anxiety in young people? (RETHINK): a randomized controlled prevention trial
Source: Trials. 2023 Apr 25;24:295. doi: 10.1186/s13063-023-07295-z (PMC10129320; doi:10.1186/s13063-023-07295-z)
Supplement: Supplementary file 1 — Additional file 1. [file 13063_2023_7295_MOESM1_ESM.docx]

**Supplementary Material - modules and key elements of the app-based interventions**

Here, we describe the different modules and key elements of the full RNT-focused intervention and the concreteness training intervention. For an overview see Table 1 in the protocol paper. As described in section 11a of the protocol paper, participants receive pop-up reminders to complete the different challenges and use the different tools in the app.

- 1. **Module: Identifying triggers of RNT and stress**
  2. **Challenge: Personal warning signs**

In this exercise, participants are guided to notice, when, where and under which conditions they tend to experience stress and engage in RNT. The app asks them to indicate typical triggers in terms of situations, times, places, bodily sensations, behaviors, and thoughts. Participants can select common triggers from a list of options and can add triggers that they cannot find in the list. The personal triggers are saved in the app so that participants can look at that at a later timepoint. The challenge is included in both the full RNT-focused intervention and in the concreteness training intervention.

- 1. **Mood tracker**

In this section of the app, participants can log their current mood, track to what extend they currently engage in RNT and indicate what they are doing at the moment. This momentary assessment option is available only in the full RNT-focused intervention.

- 1. **Concreteness Training**

**2.1 Challenge: Abstract versus concrete thinking**

This exercise guides participants to compare what effects abstract RNT versus an opposing concrete processing style has on them. To make it more tangible, participants first listen to an audio file of a fictive ambiguous interpersonal situation (waiting for a friend in a café but the friend does not show up). They are instructed to imagine themselves being in this situation. Consequently, participants are asked to think about this situation in an *abstract* manner. The induction of the abstract thinking style involves over general questions about potential reasons, consequences or meanings of the situation (Why does this always happen to me? What does this mean about our friendship? What are the consequences?). In the second part, the audio guide instructs participants to think about the same fictive scenario in a concrete manner. The concrete thinking induction includes questions about how the situation unfolded, specific sensations and emotions associated with the situation as well as concrete solutions (How did the situation unfold step by step, which specific emotions and sensations does it make them feel, what are first steps to take action?). Finally, the app asks participants to compare abstract and concrete thinking – in which condition did they feel better, what lead to better solutions, what were the main differences? The challenge is included in both the full RNT-focused intervention and the concreteness training intervention.

**2.2. Tool: Concrete thinking**

In this exercise, an audio guide helps participants to apply concrete thinking to a difficult situation which they personally experienced, either a recent event or a situation from their past. The tool is included in both the full RNT-focused intervention and the concreteness training intervention.

- 1. **Opposite Action**

**3.1. Tool: Opposite Action**

In this exercise, participants are asked to indicate which negative emotions they currently feel (e.g., sadness, anxiety, anger). Instead of engaging in RNT as a response, they are guided to act opposite the emotion. The app instructs the participant to try out facial expressions, bodily postures and behaviors that are contrary to the emotion which they are currently feeling. These opposite actions can be selected from a list of options or be added individually. The exercise is only available in the full RNT-focused intervention.

- 1. **Self-Compassion**

**4.1. Challenge: Kind versus unkind self-talk**

This exercise guides participants to compare, what effects unkind self-talk (that is typical for RNT) versus kind self-talk has on them. An audio guide first instructs participants to remember a situation in which they experienced failure and were critical of themselves. Participants are asked to vividly remember how they talked to themselves in a critical way (what did they say to themselves, how did their voice sound like, what did this critical self-talk make them feel?). Then, the audio guide instructs them to imagine that the same situation happened to a good friend. The audio guide asks participants to think about what they would say to their friend to cheer them up. Consequently, participants are guided to apply this same way of kind and encouraging talk to themselves. Finally, the app asks participants to compare unkind and kind self-talk – in which condition did they feel better, what lead to better solutions, what were the main differences? The challenge is only available in the full RNT-focused intervention.

**4.2 Tool: Kind self-talk**

In this exercise, an audio guide helps participants to apply kind self-talk to a difficult situation which they personally experienced, either a recent event or a situation from their past. The tool is only included in the full RNT-focused intervention.

- 1. **Mindfulness**

**5.1 Tool: Mindfulness**

In this section of the app, participants can try out different mindfulness exercises as a way to break repetitive thought cycles, i.e., progressive muscle relaxation, a being present in the here and now mediation and a body scan mediation. All mindfulness exercises are administered via audio files. The tool is only included in the full RNT-focused intervention.

- 1. **Setting Priories**

**6.1: Tool: Setting Priories**

In this exercise, participants are guided to select the most relevant tasks from their current tasks and make plans about how to tackle them as a strategy to stop thoughts spiraling from one task to the next. The tool is only included in the full RNT-focused intervention.

- 1. **Transfer to everyday life**

**7.1 If-then-plans**

In this section, participants can make if-then-plans. The goal is to set a specific action plan for when experiencing triggers of RNT. For example, “*If* a situation does not go as planned and ruminate about what I did wrong, *then* I will use the tool kind self-talk and try to treat myself as I would treat a good friend.” The if-then-plans are saved in the app so that participants can look at them at a later time point. After learning new strategies in the app (as part of the tools or challenges), participants are encouraged to include these in their if-then-plan. The section if-then-plans is available in both the full RNT-focused intervention and the concreteness training intervention.
